# Thalamic Circuit Mechanisms Link Sensory Processing in Sleep and Attention

**DOI:** 10.3389/fncir.2015.00083

**Published:** 2016-01-05

**Authors:** Zhe Chen, Ralf D. Wimmer, Matthew A. Wilson, Michael M. Halassa

**Affiliations:** ^1^Department of Psychiatry, NYU Langone Medical CenterNew York, NY, USA; ^2^Department of Neuroscience and Physiology, NYU School of MedicineNew York, NY, USA; ^3^The Neuroscience Institute, NYU School of MedicineNew York, NY, USA; ^4^Picower Institute for Learning and Memory, Massachusetts Institute of TechnologyCambridge, MA, USA; ^5^Center for Neural Science, New York UniversityNew York, NY, USA

**Keywords:** thalamic reticular nucleus, thalamic inhibition, attention, sleep spindles

## Abstract

The correlation between sleep integrity and attentional performance is normally interpreted as poor sleep causing impaired attention. Here, we provide an alternative explanation for this correlation: common thalamic circuits regulate sensory processing across sleep and attention, and their disruption may lead to correlated dysfunction. Using multi-electrode recordings in mice, we find that rate and rhythmicity of thalamic reticular nucleus (TRN) neurons are predictive of their functional organization in sleep and suggestive of their participation in sensory processing across states. Surprisingly, TRN neurons associated with spindles in sleep are also associated with alpha oscillations during attention. As such, we propose that common thalamic circuit principles regulate sensory processing in a state-invariant manner and that in certain disorders, targeting these circuits may be a more viable therapeutic strategy than considering individual states in isolation.

## Introduction

Impaired sleep is a complaint that is widely encountered in clinical practice, encompassing disorders that are primarily brain-based and others that are not (Zisapel, 2007). Because sleep is restorative of brain function (Siegel, 2009; Xie et al., 2013), it is often targeted by hypnotics (Walsh et al., 1995) and behavioral therapy (Edinger et al., 2001; Martínez et al., 2014), with the idea that rescuing sleep will lead to general improvement in cognition regardless of disease etiology. Targeting sleep, while undoubtedly helpful in neurodevelopmental disorders like schizophrenia and autism (Cortese et al., 2013; Wamsley et al., 2013), may be insufficient for cognitive enhancement. Sleep disruption in such disorders is seen in the context of impaired attention (Neumann et al., 2006), executive function (Xu et al., 2014) and working memory (Collins et al., 2014; Kaller et al., 2014), but this comorbidity may be the result of dysfunctional circuits normally required for sleep and cognition, rather than impaired sleep causing cognitive deficits. In support of this notion, studies have shown that both schizophrenia and autism exhibit reduction in fast electrical rhythms known as sleep spindles; phasic 7–15 Hz oscillations seen in surface electroencephalographic (EEG) recordings during sleep (Limoges et al., 2005; Ferrarelli et al., 2010). Spindles are generated by interactions between thalamic reticular nucleus (TRN) neurons and thalamo-cortical relay neurons. The TRN is a group of GABAergic neurons that surround thalamic relay neurons and provide them with a major source of inhibition (Steriade et al., 1985, 1987; Pinault, 2004; Halassa et al., 2011). The TRN is also known to be involved in attentional processing (McAlonan et al., 2006; Wimmer et al., 2015), and manipulating the TRN causes changes in both attention (Halassa et al., 2014; Ahrens et al., 2015; Wimmer et al., 2015) and sleep (Kim et al., 2012). As such, it would be reasonable to speculate that perturbed TRN circuits lead to correlated disruptions in sleep and attention. Turning this speculation into a testable model requires knowing the degree of overlap between TRN circuits that are engaged in sleep and those that are engaged in attention. That is, to what extent does spindle-generating circuitry engage in attentional processing? In this study, we attempted to answer this question by examining electrophysiological activity of identified TRN neurons across these two behavioral states. Surprisingly, not only did we find that spindle-generating TRN circuits engage in attentional processing, but we also discover that this engagement involves alpha oscillations, a waking rhythm with computational properties similar to spindles. These findings support a model in which common thalamic circuits regulate sensory processing across sleep and attention, and suggest that targeting these circuits or their computational principles may be an effective therapeutic strategy in neurodevelopmental disorders.

## Methods

VGAT-ChR2 mice were obtained from the Jackson Labs and maintained on a C57Bl6/J background. VGAT-Cre mice were backcrossed to C57Bl6/J mice for at least six generations. All experiments were conducted according to the guidelines of the Institutional Animal Care and Use Committee (IACUC) at Massachusetts Institute of Technology (MIT), the New York University Langone Medical Center and the US National Institutes of Health.

### Virus injections and drive implantations

For all surgical procedures, VGAT-cre mice were anesthetized with 1% isoflurane and mounted on a stereotactic frame. To transfect visually connected TRN neurons with channelrhodopsin 2 (ChR2), 0.5–0.8 μl of pseudotyped retrograde lenti-virus (lenti-EF1α-DIO-ChR2-EGFP, Halassa et al., 2014) were injected into the lateral geniculate nucleus (LGN; from bregma: AP, −2.1 mm, ML, 2 mm, DV, −2.5 mm). For electrophysiological experiments, drive implants with 12 independently adjustable microdrives carrying 1-2 stereotrodes [12.5 micron nichrome or 25 micron tungsten (California Fine Wire Company, Grover Beach, CA)] and a fixed optical fiber (Doric Lenses, Quebec, Canada) targeting caudal TRN were used (Halassa et al., 2014). After drilling a 2–3 mm craniotomy (center coordinates: AP, −2 mm, ML, 2.5 mm) followed by a durotomy, the implant was attached to a stereotaxic arm and lowered in a 15° angle (relative to midline) until electrodes penetrated the cortex (< 500 microns). Three stainless steel screws were implanted as EEG (prefrontal location and cerebellar reference) and ground (cerebellar). Two wires (A-M systems, Carlsborg, WA) were inserted into the neck muscle to serve as EMG. Dental cement was used to fix the implant to the skull. Mice were allowed to recover for at least one week before recordings began.

### Electrophysiology and recording

Upon recovery of implant surgery, each animal was connected to a custom made 32-channel preamplifier headstage (Neuralynx). A recording session consisted of 1–2 h behavioral testing followed by 1–2 h of post-behavioral sleep recording, allowing for neuronal activity analysis both during sleep and wakefulness. All data were recorded using a Neuralynx Digilynx recording system. Signals from each stereotrode were amplified and filtered between 0.1 Hz and 9 kHz and digitized at ~30 kHz. LFPs were collected from a single channel on each stereotrode and chosen based on recording quality (absence of low-frequency noise and movement artifacts). Stereotrodes were slowly lowered (over several days) in 125–250 micron steps to collect spike activity. Spike sorting was performed offline using the MClust toolbox (http://redishlab.neuroscience.umn.edu/mclust/MClust.html), based on spike amplitudes and energies on the two electrodes of each stereotrode. Units were separated by hand, and cross-correlation and autocorrelation analyses were used to confirm unit separation. The stability of the neuronal activity over time was visually verified (see examples in Supplementary Figure 1). Identification of TRN units was described previously (Wimmer et al., 2015). For identification of optogenetically-tagged visual TRN neurons, a fiber optic patch cord (Doric Lenses) delivered light from a 473 nm laser (Opto Engine) to the fiber optic connector on the animal's implant. Prior to connecting to the animal, laser power was measured and titrated to ~10 mW using a neutral density filter (Thorlabs). Power at the tip of the implanted fiber was ~50% of this value, based on measurements prior to surgery. Thus, there was 4–5 mW of power at the fiber tip, or 140–180 mW mm^−2^ for a 200-micron fiber. An analog stimulus generator was used to control laser pulses of 10 ms duration and 0.01 Hz frequency.

### Visual detection task

We trained mice on a visual detection task that required attentional engagement. Animals were food restricted to and maintained at 85–90% of their *ad libitum* body weight. Body weight was monitored daily and the amount of regular mouse chow (LabDiet, St. Louis, MO) was adjusted based on the number of rewards the mice received during behavioral training and testing. Experiments were conducted in a standard modular test chamber (Med Associates, St. Albans, VT). The chamber was modified to form an isosceles triangle: 23 × 24 cm (base × height). The front wall contained two white light emitting diodes, 6.5 cm apart, mounted below two nose-pokes. A third nose-poke with response detector was centrally located on the grid floor, 6 cm away from the base wall and two small Plexiglas walls (3 × 5 cm), opening at an angle of 20°, served as a guide to the poke. All nose-pokes contained an infrared LED/infrared phototransistor pair for response detection. At the level of the floor-mounted poke, two headphone speakers were introduced into each sidewall of the box, allowing for sound delivery. Trial logic was controlled by custom software running on a microcontroller. Liquid reward consisting of 10 μl of evaporated milk was delivered directly to the lateral nose-pokes via a single-syringe pump.

A white noise auditory stimulus signaled the opportunity to initiate a trial. Mice were required to hold their snouts for 500–700 ms into the floor mounted nose-poke unit for successful initiation (stimulus anticipation period). In a few sessions, holding time was increased up to 900 ms to investigate the impact of a prolonged anticipatory window. Following initiation, a stimulus light (500 ms) was presented either to the left or to the right. Responding at the corresponding nose-poke resulted in a liquid reward (10 μl evaporated milk) dispensed directly at the nose-poke.

### Optogenetic inactivation experiments

Following successful training on the visual detection task, three VGAT-ChR2 mice were anesthetized with 1% isoflurane and implanted with 3–5 mm long optic fibers (Doric Lenses, Quebec, Canada) targeting bilateral LGN (AP, −2.1, ML, ±2, DV, −2.1) and primary visual cortex (V1: AP, −3.5, ML, ±2.5, DV, −0.5). For determining visual detection psychometric function, visual stimulus duration was 0.1 s and the light was randomly displayed at one of five different intensities (0.15, 0.3, 0.6, 0.9, 1.2 lumens). For LGN or V1 inactivation, laser trains of blue light (50 Hz, 18 ms pulses, 90% duty cycle) at an intensity of 8 mW were delivered bilaterally on every other trial during stimulus presentation. Optogenetic drive of inhibitory neurons in the ChR2 mouse suppresses activity in the excitatory neurons (Zhao et al., 2011) and has been previously used to silence cortical and thalamic regions (Halassa et al., 2011; Guo et al., 2014; Wimmer et al., 2015). Performance was assessed based on the fraction of correct responses relative to chance level (50%, γ). Visual detection threshold (α) and maximum performance (λ) were estimated by fitting performance across stimulation intensities with a logistic function:

$$\begin{array}{l} F\left(x;\alpha ,\beta ,\lambda ,\gamma \right)=\gamma +\frac{\left(1-\gamma -\lambda \right)}{1+\text{exp}\left(-\beta \left(x-\alpha \right)\right)} \end{array}$$

where *x* corresponds to the five stimulus intensity levels expressed as a percentage of maximum stimulus intensity. The fraction of correct trials was summed across sessions and the overall performance as a function of stimulus intensity was fit using maximum likelihood estimation implemented in the Palamedes psychophysical toolbox (http://www.palamedestoolbox.org/). Estimation of α was made via non-parametric bootstrap analysis of curve fits.

### Analysis of vigilance state dependent firing

Unit activity during the different vigilance states was determined as previously described (Halassa et al., 2014). Based on simultaneous EEG and EMG recordings, behavioral epochs were classified into three states: wake, slow-wave sleep (SWS), and rapid eye movement (REM) sleep. Wake epochs were identified by high EMG activity, and the REM epochs were determined by a low EMG activity and high EEG theta/delta power ratio. The remaining epochs were treated as SWS epochs and scoring was visually verified. Minimum criteria for wake and SWS were >16 s and REM was >5 s. Activity of individual TRN neurons was determined for each vigilance state by calculating the firing rate with 1 s bin size and computing the mean of all instantaneous binned firing rates of the same state. Cells with firing rate ratio wake/SWS >1 were considered to be wake-active, and cells with ratio wake/SWS < 1 was considered to be sleep-active.

### Spectral analysis

Multitapered spectral analyses were performed using the Chronux toolbox (www.chronux.org; Mitra and Bokil, 2008), which included the LFP spectrogram and spike-field coherence (SFC). The SFC measures phase synchronization between the LFP and spike times as a function of frequency. In all analyses, to reduce the estimation bias we used the multi-taper method instead of an arbitrary windowing method. Specifically, we chose a half-bandwidth parameter *W* such that the windowing functions are maximally concentrated within [−*W, W*]. We chose *W* > 1/*T* (where *T* denotes temporal duration) such that the Slepian taper functions are well concentrated in frequency and have bias reducing characteristic (Bokil et al., 2010). In terms of Chronux function setup, we used the tapers setup [*TW K*], where *TW* = 3 is the time-bandwidth product, and *K* = 2*TW-*1 = 5 is the number of tapers. In addition, since the taper functions are mutually orthogonal, they give independent spectral estimates. In turn, by reducing one taper one can compute robust, Jackknife-based confidence intervals on all estimates, even for a single trial. In the visual detection task, we computed SFC at both in-attention window (0 to the first 1 s after trial initiation, [0, 1] s) and outside-attention window (10–11 s after trial initiation, [10, 11] s). In all time-frequency analyses, we used a window size of 500 ms (which covers at least 4 alpha cycles) and a moving window step size of 10 ms.

### Spike-phase synchrony analysis

We applied a Hilbert transform to compute an analytic signal and its instantaneous phase value for the LFP. We filtered the LFP within a specific frequency band of interest, e.g., the alpha band (9–15 Hz). For each TRN unit, we constructed a circular spike-phase histogram (24 bins within 360°) and measured the spike-LFP phase synchrony in two steps. First, a neuron was considered phase locked only if the distribution of the spike phase angles departed from a uniform circular distribution (Rayleigh's test for circular uniformity, *P* < 0.05). We used a standard and well-established circular statistics MATLAB toolbox (MathWorks Inc., Natick, MA) for statistical analyses (Berens, 2009). TRN cells with firing rate below 1 Hz were excluded in this analysis. Next, to further quantify the degree of spike phase locking, we applied an inverse cosine transformation to the spike count vector derived from the spike phase histogram (Halassa et al., 2014), and computed the Pearson correlation statistic from the sample scatter plot. A high degree of correlation indicates a well fit of the cosine phase tuning of spike data or high spike-phase synchrony. *P* < 0.05 was considered to be statistically significant.

### Pairwise neuronal spike-time synchrony analysis

**Figure 1:**
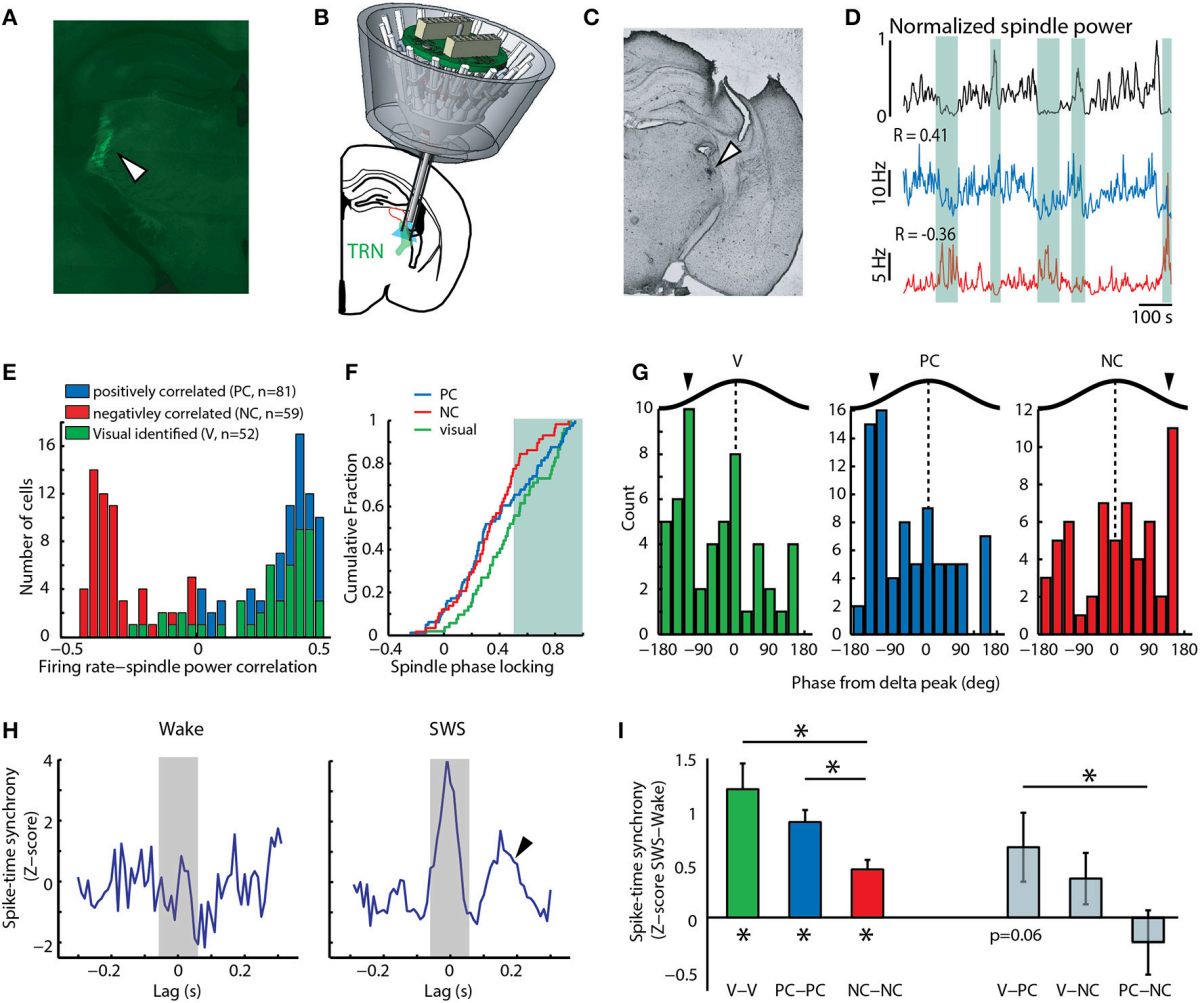
**Positive correlation to spindles among TRN neurons predicts involvement in sensory processing. (A)** Histological section showing Cre-dependent retrograde tagging of visTRN. **(B)** Cartoon depiction of multi-electrode implant with optical fiber targeting caudal TRN, where visTRN neurons are located. **(C)** Histological verification of TRN targeting in one mouse. **(D)** Examples of two untagged TRN neurons displaying positive (blue) and negative (red) correlation to spindle power (black). Shaded areas highlight portions of high or low spindle power and the opposing changes in firing rate between the positive and negative correlated example neurons. **(E)** Population data reveals a bimodal distribution of rate-spindle power correlation among TRN populations (PC, *n* = 81; NC, *n* = 59), with visTRN neurons (*n* = 52) exhibiting a predominantly positive correlation. **(F)** visTRN neurons show higher spindle phase locking values compared to NC neurons. PC neurons are more visTRN-like at higher phase-locking values (shaded area). **(G)** visTRN and PC neurons show similar phase preferences to delta waves in sleep, which is opposite to NC neurons. **(H)** Example spike-time synchrony (Z-score) plot from a PC neuronal pair, showing elevated values in SWS compared to wake. Only the integral of the correlation value [−50, 50] ms (shaded area) was taking into account for quantification. Arrow head denotes second peak at 200 ms, suggestive of delta entrainment. **(I)** Group data of quantifying phenomenon in panel h; note that visTRN and PC populations exhibit the highest synchrony values, and both are significantly different than NC neurons (^*^P < 0.05, Wilcoxon rank-sum test). When comparing across group, visTRN and PC heterologous pairs are the only ones that show a value of marginal significance (*P* = 0.06, Wilcoxon signed-rank test).

We computed the spike-time synchrony between paired TRN cells during sleep and awake states (time lag [−500, 500] ms, bin size 10 ms). We then computed the mean and SD of the cross-correlation profile (which is non-negative and non-symmetric). The correlation value above or below 2 SD was considered significant. We integrated the significant correlation value in a small window ([−50, 50] ms, shaded area of Figure 1H) and computed the averaged Z-score as a measure of synchrony. Positive Z-score indicates an excitatory effect from the trigger cell to the target cell, whereas negative Z-score indicates an inhibitory effect. All comparisons in Figure 1I failed the test for normality (Anderson-Darling test, V-V *n* = 46 pairs, *P* < 1e-5, PC-PC *n* = 144 *P* < 1e-5, NC-NC *n* = 164, *P* < 1e-5, V-PC *n* = 108, *P* < 1e-5, V-NC *n* = 108, *P* < 1e-5, PC-NC *n* = 131, *P* < 1e-5) and were compared using Wilcoxon rank-sum test.

### Group average spike phase modulation

We applied a Hilbert transform to compute an analytic signal and its instantaneous phase value (MATLAB function “hilbert”) for the local LFP. During sleep or attention, we band-passed the LFP within the spindle or alpha frequency band (9–15 Hz). For each TRN unit, we constructed a spike-phase histogram (within 0–360°), which measures the spike phase modulation (SPM). For each TRN cell group (either visTRN, PC or NC), we computed the weighted mean and weighted standard deviation (SD) of the group SPM according to the number of spikes of each cell (**Figure 4B**). For instance, let *N*_*i*_ denote the number of spike from the *i*-th cell, and let *SPM*_*i*_ denote the SPM curve computed from the spike-phase histogram spike from the *i*-th cell, the group mean and SD are computed as (Halassa et al., 2014)

$$\begin{array}{lll} {SPM}_{\text{mean}} & = & \frac{\underset{i}{\sum }N_{i}{SPM}_{i}}{\underset{i}{\sum }N_{i}},\\ {SPM}_{\text{SD}} & = & \sqrt{\frac{\underset{i}{\sum }N_{i}{\left({SPM}_{i}-{SPM}_{\text{mean}}\right)}^{2}}{\underset{i}{\sum }N_{i}}} \end{array}$$

### Statistical analysis

All data with *n* ≥ 50 was tested for normality using Anderson-Darling test. For data that were non-normal, unipolar (e.g., data are all positive) or of small sample size, non-parametric statistics were used. When comparing two odds ratios from two independent sample groups, we first computed the sample proportions *p*_1_ and *p*_2_ based on sample sizes *n*_1_ and *n*_2_. The null hypothesis H_0_ is assumed to be *p*_1_ = *p*_2_. We then computed the *z*-score using the formula

$$\begin{array}{l} z=\frac{p_{1}-p_{2}}{\sqrt{\frac{p_{1}\left(1-p_{1}\right)}{n_{1}}+\frac{p_{2}\left(1-p_{2}\right)}{n_{2}}}} \end{array}$$

where the denominator denotes the standard error (SE). The confidence intervals (CIs) for the difference of two odds are (*p*_1_ - *p*_2_) ± *z* SE. Then the one-sided or two-sided *P*-valued associated with the *z*-value can be computed (*z* = 1.96 for a 95% CI and *z* = 2.58 for a 99% CI). We reject the null hypothesis H_0_ if *P* < 0.05.

To determine neurons with significant firing rate modulation, we computed the mean firing rate during the task period (FR_task) and baseline (FR_baseline) at each trial, where the task period is defined within [0, 0.5] s after trial initiation and baseline is defined as 2.5 s before trial initiation. Then the relative firing rate change was calculated:

$$\begin{array}{l} \text{Reliatve rate change}=\;\frac{\text{mean of FR\_task }-\text{ mean of FR\_baseline}}{\text{ mean of FR\_baseline}} \end{array}$$

A neuron was considered to be significantly modulated if two conditions were satisfied: First, the absolute value of the Relative rate change was >0.2. Second, the difference between the matched samples in the vectors FR_task and FR_baseline came from a distribution whose median was non-zero (two-sided Wilcoxon signed-rank test).

## Results

To begin investigating the overlap between sleep and attention microcircuits in the TRN, we analyzed a previously collected electrophysiological recordings dataset containing activity of identified TRN neurons in freely behaving mice (Halassa et al., 2014). In each recording session, mice were run on an attention-requiring detection task, after which they were allowed to sleep. Based on previous analysis, we concluded that the TRN is composed of functional subnetworks defined by anatomical connectivity, and that this architecture allows the TRN to independently control its thalamic targets in a behaviorally relevant manner (Halassa et al., 2014). We focused our analysis here on TRN neurons that are connected to visual thalamus (visTRN), which we had anatomically and physiologically identified by retrograde, Cre-dependent optogenetic tagging (Figures 1A–C, Supplementary Figure 2). Similar to results previously obtained from rostral TRN, we found that during slow wave sleep (SWS), this caudal region contained untagged neurons whose firing rates were positively correlated (PC) and others that were negatively correlated (NC) to spindle power (Figures 1D,E). The majority of visTRN neurons (44/52) tended to be PC to this measure, and overall, these neurons showed higher phase-locking to locally-recorded spindles than NC neurons (Figure 1F). Interestingly, PC neurons showed a mixed distribution, approaching visTRN neurons at higher phase locking values (Figure 1F). These findings confirm earlier results that had proposed a sensory origin for spindles (Sato et al., 2007; Timofeev and Chauvette, 2013; Halassa et al., 2014), and raise the possibility that at least a proportion of PC neurons are sensory in nature. The PC group might include a small fraction of unlabeled visTRN neurons. However, in contrast to the 90% of optogenetically tagged visTRN neurons that responded to full field visual stimulation, only 18% of PC neurons were visually responsive. This small potential misclassification cannot account for the fact that 40% of PC neurons exhibited spindle phase locking values equivalent to visTRN.

If PC neurons contained a sensory population, we would expect them to share physiological features with visTRN, and that these features would be indicative of engagement in sensory processing. During SWS, PC neurons showed three features that were “sensory-like.” First, PC and visTRN neurons showed a preference to the same delta (0.5–4 Hz) phase (Figure 1G, *P* < 0.001, binomial test), suggesting that they are entrained by similar cortical connectivity mechanisms (Slézia et al., 2011; Sheroziya and Timofeev, 2014). Second, analysis of vigilance state dependent firing revealed that visTRN and PC neurons had a substantially larger proportion that was “sleep-active” than NC neurons (vis: 23/52, PC: 35/81, NC: 7/59; *P* < 0.01, two-sample proportion test), suggesting similar mechanistic regulation by arousal states (Halassa et al., 2014). Third, while all caudal TRN neurons showed enhanced spike-time synchrony during SWS compared to wake (Figure 1H and left side of Figure 1I), PC and visTRN showed higher values compared to NC neurons (*P* < 0.01, Wilcoxon rank-sum test). SWS synchrony among pairs containing one visTRN and one PC neuron reached marginal significance (*P* = 0.06, Wilcoxon singed-rank test), compared to values obtained from other heterologous pairs, which were all non-significant (right side of Figure 1I). The higher proportion of sleep-active cells and the generally more robust sleep synchrony within and across visTRN and PC neurons suggest that these populations provide more inhibition to their thalamic targets compared to NC neurons. This is supportive of the notion that, just like visTRN, PC neurons are involved in sensory processing, perhaps by rendering their targets less responsive to sensory stimuli during sleep.

**Figure 2:**
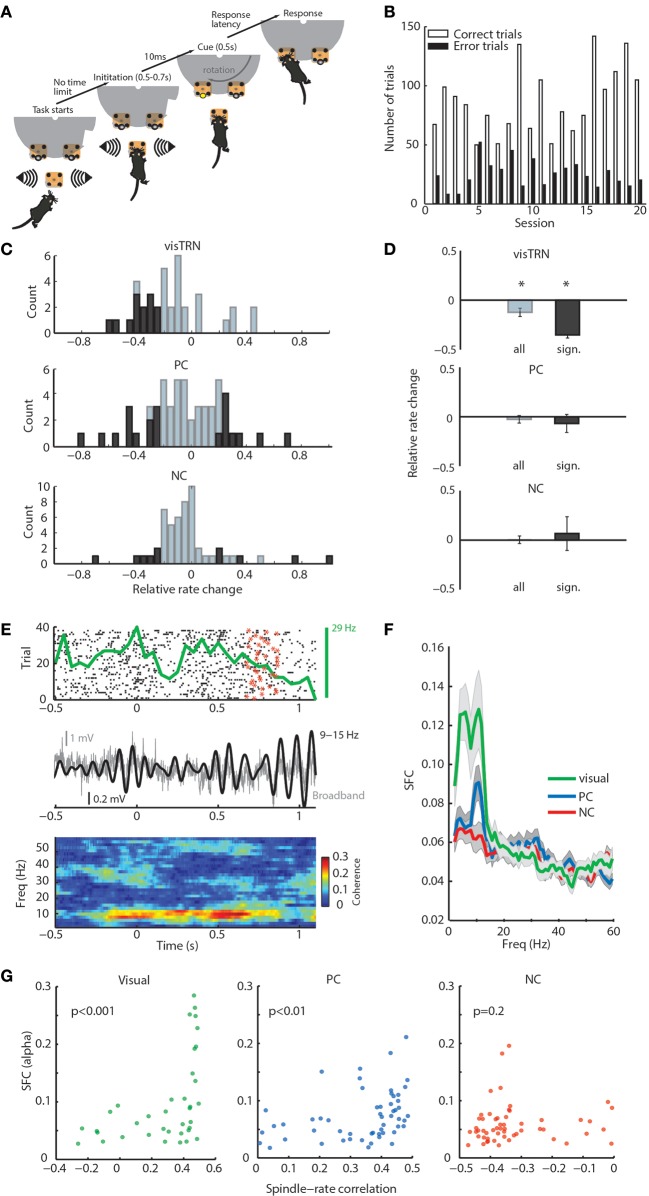
**General and modality-specific sensory engagement of TRN neuronal populations. (A)** Schematic diagram of visual detection task. **(B)** Task performance showing the proportion of correct and incorrect trials for each of the 20 recorded sessions. **(C)** Distribution of rate modulation values of TRN neurons in the stimulus anticipatory period of the task shown separately for the three groups (visTRN, *n* = 37; PC, *n* = 55; NC, *n* = 55). Dark colors denote significance per cell (see statistical criterion in Methods). Note that all significant visTRN neurons show negative modulation; PC neurons, while showing a comparable number of neurons, are split and NC neurons show very few significantly modulated neurons. **(D)** Grouped data for panel **(C)**. ^*^P < 0.05 (one-sided Wilcoxon signed rank test). Only visTRN neurons show a significant decrease in firing rate, consistent with increased gain in visual thalamic sensory processing. **(E)** Example showing a positive correlation between a visTRN neuronal rate (spike raster: black dots; star denotes the offset of anticipation period at each trial; PSTH: top green curve), and alpha amplitude (middle black curve) during the task (0 denotes trial initiation). This positive correlation is reflected in alpha spike-field coherence (SFC) observed during stimulus anticipation and presentation (bottom). **(F)** alpha SFC specific to visTRN neurons within the anticipatory window ([0, 1] s). **(G)** alpha SFC during awake attention covaries significantly with spindle-rate correlation in visTRN (*P* < 0.001, ρ = 0.6) and PC (*P* < 0.01, ρ = 0.42), but not NC neurons (*P* = 0.2, ρ = 0.17; *P*-values denote significance for Spearman's rank correlation).

To further examine sensory-related features across the three TRN populations, we analyzed their activities in a visual detection task, as had been previously described (Halassa et al., 2014). The task consisted of self-initiated trials, where a food deprived mouse had to appropriately position its head for 500–700 ms, after which a bright (1.2 lumens), 500 ms white light LED stimulus was presented. The location of the visual stimulus indicated the side at which the reward (20 μL of evaporated milk) was available (Figure 2A), and the mouse had 15 s to collect it. Under these conditions, the visual detection task performance is dependent on sensory processing at the level of LGN but not primary visual cortex V1, as determined by optogenetic inactivation experiments (Supplementary Figure 3). Mice performed this task with high accuracy (in 40% of the sessions, mice performed at ≥84% accuracy; 20 sessions, *n* = 2 animals, Figure 2B).

Our previous study showed that, as a population, visTRN neurons exhibited a decrease in firing rate during the stimulus anticipation period (Halassa et al., 2014). Here, we evaluated firing rate changes on a cell-by-cell basis, where the magnitude, direction and significance of change were all considered. We found that the relative rate change distribution for visTRN neurons exhibited a leftward shift (Figure 2C, top; and Supplementary Figure 4 for a representative cell). All visTRN cells showing a significant change exhibited a decrease in firing rate (Figure 2C, top). PC neurons had a comparable proportion of significantly modulated neurons as visTRN, but only 12/21 showed a decrease (Figure 2C, middle). In comparison to visTRN and PC, NC neurons showed a very small number of significantly modulated cells (Figure 2C, bottom). As a population, only visTRN neurons showed a reduction in firing rate during anticipation of a visual stimulus (Figure 2D), supporting previous findings that TRN contains modality-specific subnetworks that provide independent control over thalamic targets (Halassa et al., 2014).

We noted that the rate changes observed across visTRN neurons were correlated with changes in local field potential (LFP) power, particularly in the alpha band (9–15 Hz; Figure 2E). To quantify this observation, we examined the SFC between visTRN neuronal rates and locally recorded LFP. There was a robust increase in visTRN-alpha band SFC around stimulus anticipation and presentation (Figure 2F), yet there was no associated change in alpha power (Supplementary Figure 5). While PC neurons showed a smaller alpha SFC peak in that same period, its value did not reach significance when compared against baseline (Supplementary Figure 6). This suggests that enhanced alpha SFC is specific to visTRN during visual stimulus anticipation, and given the spectral overlap between alpha and spindles, it also suggests that TRN neurons show sensory-related dynamics that are qualitatively similar across states. In support of this conclusion, we observed a strong positive correlation between alpha-SFC in attention and firing rate-spindle correlation in sleep (Figure 2G).

**Figure 3:**
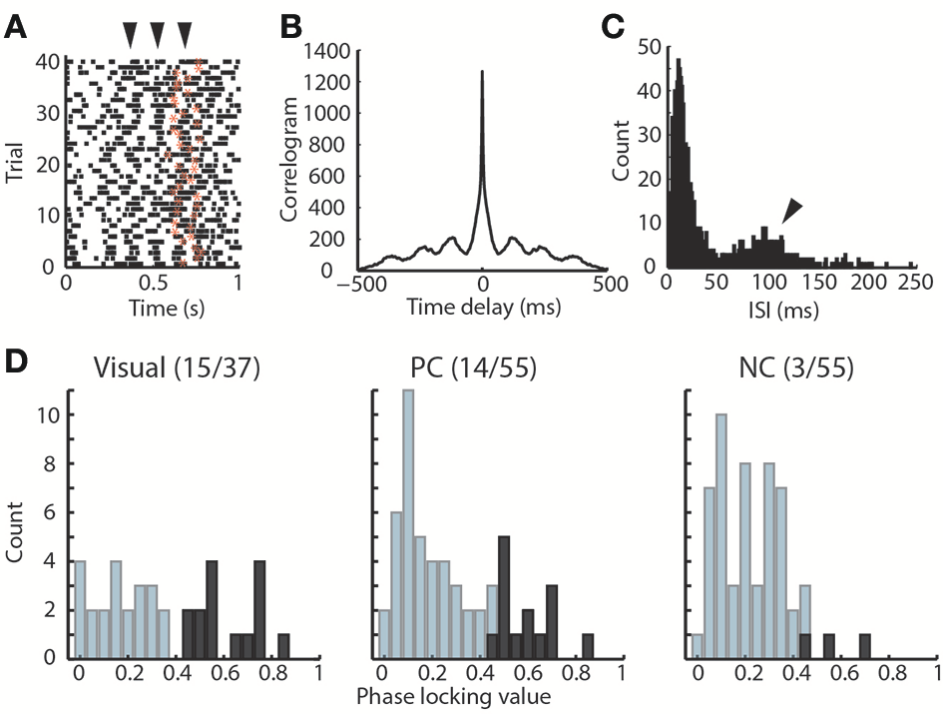
**Rhythmicity of visTRN neurons in the visual detection task. (A)** Example raster of one visTRN neuronal spikes in the task, zero denotes trial initiation and star denotes the offset of anticipation period. Note the rhythmic temporal structure occurring at 100 ms intervals (arrowheads indicate spike rhythmicity). **(B)** Autocorrelogram of this unit in the task highlighting the rhythmic structure. **(C)** Interspike interval (ISI) histogram of this unit showing a peak at 100 ms. **(D)** Histogram of phase-locking values for the three TRN populations. Dark color indicates units with significant phase-locking. Note that visTRN neurons show the largest proportion of significantly phase-locked units (15/37), followed by PC cells (14/55), while NC neurons are rarely phase-locked (3/55).

**Figure 4:**
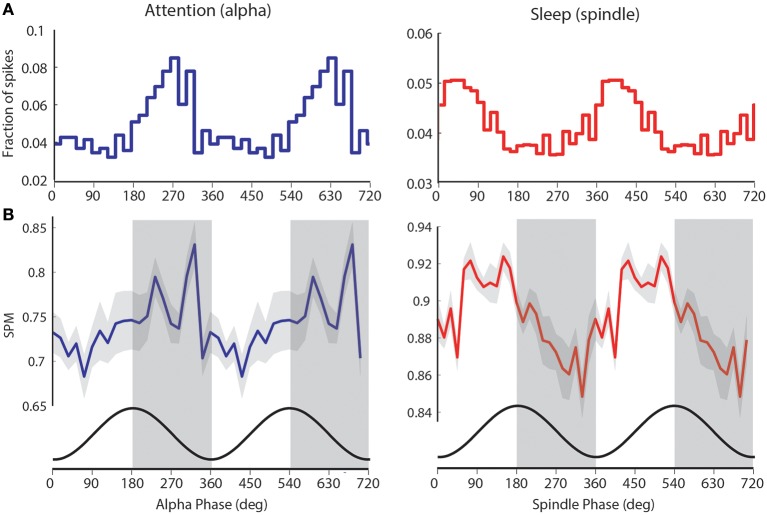
**visTRN firing during attention and spindles occurs at opposite phase of alpha. (A)** Example of visTRN neuronal alpha (blue) and spindle phase (red) histograms, showing robust phase modulation of spike times. Note the opposite phases of visTRN peak firing in attention and sleep (spindle). **(B)** Average of significantly spike-phase modulated neurons (*n* = 15) weighted by their firing rates shows a clear rhythmic structure in relationship to ongoing alpha. Cosine curve (black) at the bottom illustrates spike-phase relationship. This, we suggest, imposes rhythmic excitability changes in the thalamus (periods of high excitability are shaded in gray).

Previous data in awake cats suggested a role for thalamic inhibition related to alpha oscillation in visual perception (Lorincz et al., 2009). We reasoned that if alpha oscillation was relevant to performance, then it would have a relationship to TRN neuronal spike times, and would therefore support a role for temporally-structured thalamic inhibition in attention. We found that TRN neurons themselves exhibit rhythmicity at alpha frequency in the task (Figures 3A–C). To evaluate the relationship between TRN rhythmicity and alpha in the LFP, we examined alpha phase-locking of TRN neurons. We found that visTRN neurons showed the largest proportion of significantly phase-locked cells, followed by PC neurons, while the NC group only contained very few phase-locked neurons (Figure 3D). This finding suggested that alpha rhythmicity may reflect a temporally-matched visTRN structure, providing rhythmic inhibition to visual thalamus (LGN). Consistent with this notion, we found that visTRN neurons fired preferentially within half of the alpha cycle, with relatively fewer cells firing at 0–180° (left side of Figure 4A).

To further explore the computational significance of this temporal structure, we weighted the preferred visTRN alpha phase-distribution with the number of spikes generated by each visTRN neuron. This revealed a more robust rhythmic structure of visTRN activity in the anticipatory window (left side of Figure 4B). This particular result is consistent with the predictive role of alpha phase in visual perception suggested by human studies (Busch et al., 2009), as it proposes a model in which the TRN imposes ~50 ms windows of alternating excitability in thalamus that can explain this perceptual dependence (Figure 4B). A corresponding rhythmic process was observed with spindles during sleep, but interestingly, visTRN firing occurred at the opposite phase of spindles compared to alpha (right side of Figures 4A,B).

## Discussion

For several years, the TRN has been considered a monolithic structure where its neurons provide uniform inhibition to thalamic targets (Llinas and Steriade, 2006). This notion had been largely based on the biophysical homogeneity encountered when TRN neurons were examined in acute (Huguenard and Prince, 1992; Bal and McCormick, 1993) or anesthetized preparations (Contreras et al., 1993; but see Lee et al., 2007). By performing electrophysiological recordings of TRN neurons in a behavioral context, we discovered that these cells are physiologically heterogeneous. Our previous findings gave rise to the notion that the TRN is composed of multiple subnetworks, each defined by its projections to a distinct thalamic target (Halassa et al., 2014). As such, thalamic inhibition may be regionally controlled, in a manner that is dependent on behavioral demand. This study confirms and extends this notion along several dimensions.

First, the finding that untagged spindle-correlated TRN neurons show activity modulation consistent with them being sensory in nature confirms the notion that spindles are sensory-related dynamics (Figures 1G–I). Importantly, this finding provides a mechanistic explanation for the well-known correlation between spindle density and gating of sensory inputs during sleep (Dang-Vu et al., 2010; Wimmer et al., 2012). Sensory TRN neuronal firing rates (a correlate of spindles) will likely determine the degree of thalamic sensory throughput during sleep.

Second, the preferential and uniform modulation of visTRN neurons in the visual detection task (Figures 2C,D) provides additional and independent evidence that thalamic inhibition can be controlled in a modality-specific manner. This also confirms the notion that inhibitory control of thalamic sensory processing does not simply co-vary with general arousal, but can rather be more precisely matched to behavioral demand. The finding that the PC population included significant firing rate increases supports the idea that modalities which are not relevant to solve the task might be suppressed. In another study, we fully evaluated this idea by developing a behavior that requires modality-specific suppression of sensory inputs (Wimmer et al., 2015).

Third, while rhythmic TRN firing in relation to spindles has been observed in sleep, to our knowledge this study is the first to show a similar rhythmic engagement during an attentional state. Just as sensory TRN neuronal firing rates show broad correlation to spindle power in sleep (Figure 1E), they do so to alpha oscillations in attention (Figures 2E–F). In fact, the degree to which a neuronal firing rate is correlated to spindle in sleep predicts its correlation to alpha in attention (Figure 2G). This finding is highly significant, suggesting a common biophysical substrate for rhythmic TRN neuronal engagement across states. Such notion would be consistent with computational studies showing that TRN-thalamic microcircuits generate rhythmic oscillations at ~10 Hz (Destexhe et al., 1993). The inverse phase relationship between visTRN firing during awake alpha and sleep spindle activity (Figure 4) might indicate overlapping but non-identical mechanisms. The stronger rhythmicity of visTRN neurons observed in attention further supports the notion of modality-specific TRN engagement in sensory processing, and extends it beyond changes in firing rate.

Does TRN rhythmicity during sensory processing result in functional consequences? One can hypothesize that TRN spiking in relationship to alpha phase (Figure 4) would result in waxing and waning thalamic inhibition at the alpha frequency. This would imply that alpha oscillations would be associated with shifts in sensitivity to incoming stimuli. Meaning, at near-threshold conditions inputs that arrive at the phase associated with least inhibition would be most likely detected, while those arriving at the opposite phase would not. Interestingly, recent human psychophysics/EEG experiments have provided empirical support to this notion; alpha phase predicts the detection of near-threshold visual stimuli (Busch et al., 2009). Furthermore, the speed of alpha oscillations across individuals appears to set the limits for temporal vision (Samaha and Postle, 2015). Our data suggest that the circuits underlying temporal attention may be thalamic, and that their boundaries are set by the biophysical properties of thalamic-TRN interactions. It is important to note that our study does not show any change in alpha power during the attention task (Supplementary Figure 5), and that our findings are consistent with the role of alpha phase in attentional processing as recently observed in primates (Saalmann et al., 2012).

The question of how the brain processes sensory information across states is a long standing one in neuroscience (Lee and Dan, 2012). Here, we have shown an unexpected overlap in thalamic mechanisms of sensory processing during sleep and attention. The finding that spindles in sleep may be an alpha in attention counterpart is intriguing, and calls for more mechanistic studies of both oscillations. More generally, our data set the stage for guided investigation of how the TRN controls thalamic gain and timing, impacting sensory processing across states.

## Funding

Work was supported by the US National Science Foundation (IIS-CRCNS 1307645 to ZC), the Swiss National Science Foundation (P2LAP3 151786 to RW), the Simons, Feldstein and Sloan Foundations (MH), the NARSAD Young Investigator Award (MH) and the US National Institutes of Health (R01-MH06197 and TR01-GM10498 to MW and R00 NS078115 to MH).

## Author contributions

MH conceived and supervised all aspects of the study. RW collected all data and analyzed behavioral aspects of the data. ZC analyzed electrophysiological aspects of the data. MW supported the initial phase of the project. MH wrote the manuscript.

### Conflict of interest statement

The authors declare that the research was conducted in the absence of any commercial or financial relationships that could be construed as a potential conflict of interest.
