# Supplementary material for: Thalamic Circuit Mechanisms Link Sensory Processing in Sleep and Attention
Source: Front Neural Circuits. 2016 Jan 5;9:83. doi: 10.3389/fncir.2015.00083 (PMC4700269; doi:10.3389/fncir.2015.00083)
Supplement: Supplementary file 1 [file DataSheet1.PDF]

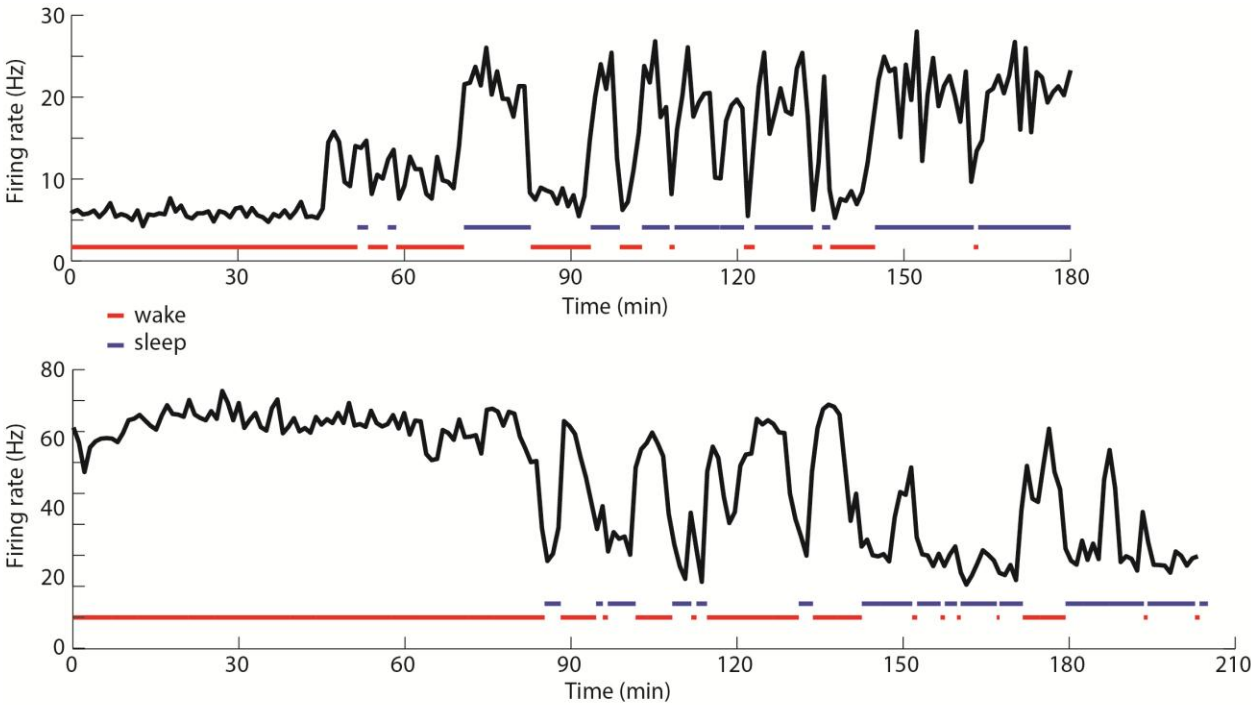

Supplementary Figure 1: Firing rate stability of neuronal activity across sleep and wakefulness. The firing rate (black trace) of a sleep-active (top) and wake-active (bottom) TRN neuron plotted across the whole duration of two different recording sessions (1 min bins). The colored bars at the bottom indicate wakefulness (red) or sleep (blue).

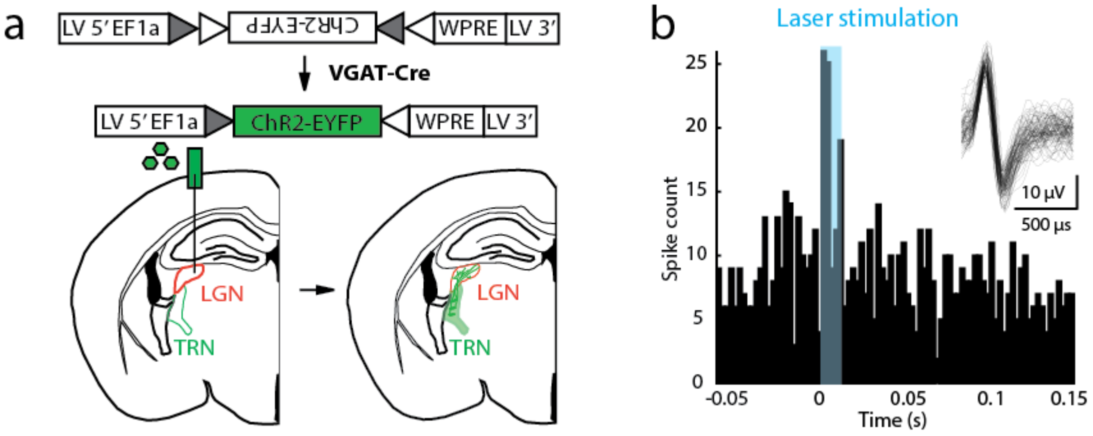

Supplementary Figure 2: Optogenetic tagging of visTRN neurons. (a) Schematic illustrating the strategy to tag visTRN neurons. A double floxed ChR2-EYFP construct packaged into a retrograde lenti-virus is injected into the LGN of a VGAT-cre mouse to retrogradely label visTRN. **(b)** Example PSTH of a TRN unit showing the spike increase in response to a 10 ms blue laser pulse. Inset displays the characteristic thin spike waveform of the TRN neuron.

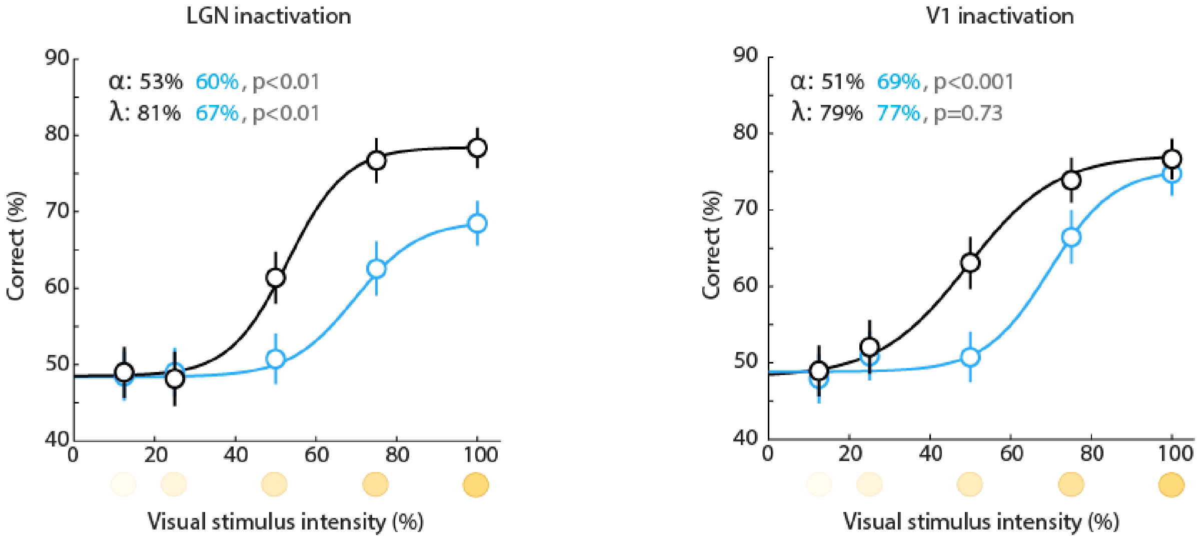

Supplementary Figure 3: Dependence of task performance on LGN and V1 sensory processing. Optogenetic inactivation experiments targeting either the lateral geniculate nucleus (LGN) or primary visual cortex (V1). Psychometric function was determined by dimming the intensity of the visual stimulus. LGN or V1 activity was disrupted by activating GABAergic terminals in the VGAT-ChR2 mouse with blue laser trains (8 mW, 50 Hz, 90% duty cycle during stimulus presentation). While both LGN and V1 inactivation shifted the detection threshold (α) to the right, i.e. towards brighter stimuli (LGN: $P < 0.01$, V1: $P < 0.001$, bootstrap comparison, n >181 trials of 3 mice), only LGN disruption reduced maximal performance (λ) at 100% stimulus intensity (LGN: $P < 0.01$, V1: $P = 0.73$, bootstrap comparison).

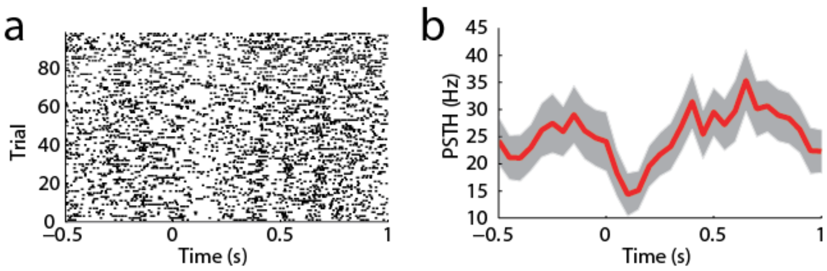

Supplementary Figure 4: visTRN cells show firing rate reduction during stimulus anticipation. (a) Raster plot and **(b)** A PSTH example of visTRN neuron showing the reduction in firing rate during stimulus anticipation (zero marks trial initiation).

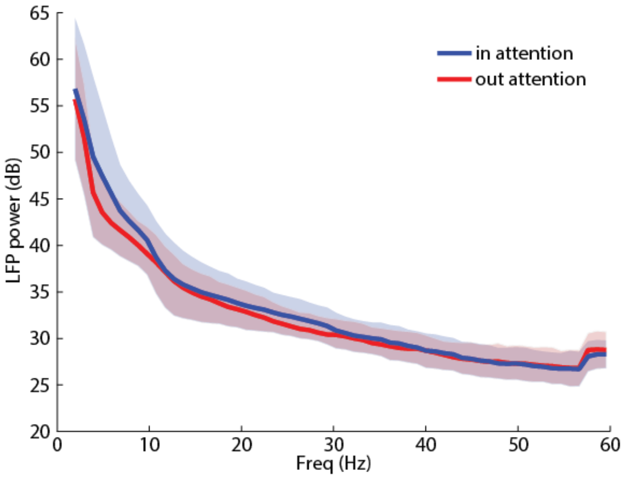

Supplementary Figure 5: LFP power inside and outside the attentional window are comparable. LFP power during the anticipatory window (0 to the first 1 second after trial initiation, [0, 1] s) was indistinguishable from that outside the anticipatory window (10-11 seconds after trial initiation, [10, 11] s).

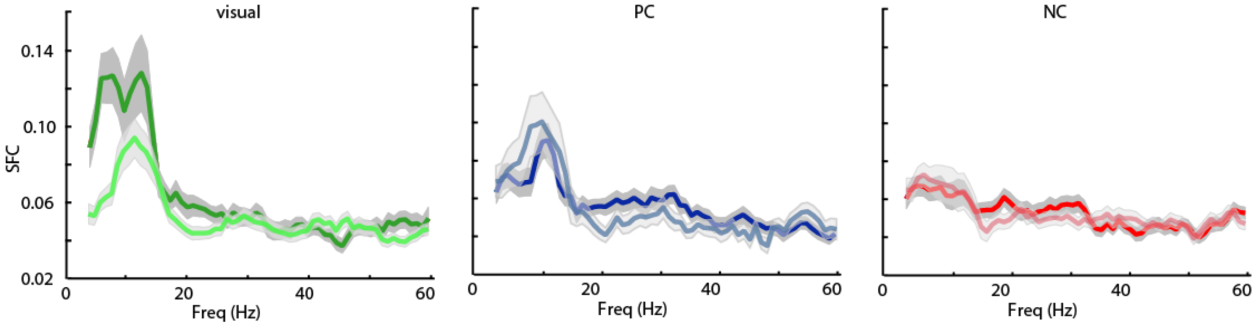

Supplementary Figure 6: Selective increase in visTRN alpha band-SFC during stimulus anticipation. visTRN and PC show higher baseline alpha-band SFC compared to NC. Only visTRN neurons show a significant elevation in the anticipatory window (0 to one second following initiation onset [0, 1] s, dark colors) compared with the outside attention window (10 – 11 s following initiation onset [10, 11] s, light colors).
